# Videos in Short-Video Sharing Platforms as Sources of Information on Colorectal Polyps: Cross-Sectional Content Analysis Study

**DOI:** 10.2196/51655

**Published:** 2024-10-29

**Authors:** Jia-Lun Guan, Su-Hong Xia, Kai Zhao, Li-Na Feng, Ying-Ying Han, Ji-Yan Li, Jia-Zhi Liao, Pei-Yuan Li

**Affiliations:** 1 Department of Gastroenterology Tongji Hospital, Tongji Medical College Huazhong University of Science and Technology Wuhan China; 2 Department of Gastroenterology Wenchang People's Hospital Wenchang China

**Keywords:** colorectal polyps, short videos, health information, quality assessment, reliability

## Abstract

**Background:**

Short videos have demonstrated huge potential in disseminating health information in recent years. However, to our knowledge, no study has examined information about colorectal polyps on short-video sharing platforms.

**Objective:**

This study aimed to analyze the content and quality of colorectal polyps-related videos on short-video sharing platforms.

**Methods:**

The terms “肠息肉” (intestinal polyps) or “结肠息肉” (colonic polyps) or “直肠息肉” (rectal polyps) or “结直肠息肉” (colorectal polyps) or “大肠息肉” (polyps of large intestine) were used to search in TikTok (ByteDance), WeChat (Tencent Holdings Limited), and Xiaohongshu (Xingyin Information Technology Limited) between May 26 and June 8, 2024, and then the top 100 videos for each search term on different platforms were included and recorded. The *Journal of American Medical Association* (JAMA) score, the Global Quality Scale (GQS), the modified DISCERN, and the Patient Education Materials Assessment Tool (PEMAT) were used to evaluate the content and quality of selected videos by 2 independent researchers. SPSS (version 22.0; IBM Corp) and GraphPad Prism (version 9.0; Dotmatics) were used for analyzing the data. Descriptive statistics were generated, and the differences between groups were compared. Spearman correlation analysis was used to evaluate the relationship between quantitative variables.

**Results:**

A total of 816 eligible videos were included for further analysis, which mainly conveyed disease-related knowledge (n=635, 77.8%). Most videos were uploaded by physicians (n=709, 86.9%). These videos had an average JAMA score of 2.0 (SD 0.6), GQS score of 2.5 (SD 0.8), modified DISCERN score of 2.5 (SD 0.8), understandability of 80.4% (SD 15.6%), and actionability of 42.2% (SD 36.1%). Videos uploaded by news agencies were of higher quality and received more likes and comments (all *P*<.05). The number of collections and shares of videos about posttreatment caveats were more than those for other content (*P*=.03 and *P*=.006). There was a positive correlation between the number of likes, comments, collections, and shares (all *P*<.001). The duration and the number of fans were positively correlated with the quality of videos (all *P*<.05).

**Conclusions:**

There are numerous videos about colorectal polyps on short-video sharing platforms, but the reliability and quality of these videos are not good enough and need to be improved.

## Introduction

Colorectal cancer (CRC) is the third most common cancer and the second cause of cancer-related death worldwide [1]. Colorectal polyps are abnormal growths and protrusions on the colorectal surface, and adenomatous colorectal polyps are the precursors of the majority of CRC [2]. With the improvement of living conditions and the changes in diet habits, the incidences of colorectal polyps and CRC are gradually increasing [3]. Most colorectal polyps are asymptomatic, and some may quietly develop into malignancies, which are more likely to be ignored. The detection and treatment of colorectal polyps is a critical way to reduce the incidence of CRC and its subsequent morbidity and mortality [4]. At the same time, lifestyle adjustments and regular examination could prevent the occurrence or recurrence of colorectal polyps [5-7]. Hence, it is necessary for the population to receive appropriate health information and self-management education.

With the development of the internet and mobile phone communication, information propagates quickly along social networks. People are increasingly seeking and accessing health information and knowledge on mobile internet. Short videos increase the efficiency of people’s scattered free time as well as the convenience of enjoyment, thus receiving wide popularity. In 2021, TikTok (ByteDance) had more than 1.6 billion monthly active users, and the number of monthly active users in WeChat (Tencent Holdings Limited) is as high as 1 billion [8,9]. At the same time, Xiaohongshu, a free social networking website, has also attracted millions of users in China in recent years [8]. On these platforms, patients can obtain a large number of health videos by searching related keywords without any cost. According to a report from Tencent, at the end of 2020, 73% of users had seen health information–related short videos and 42% of users would watch related short videos 1-3 times per week for acquiring health information or seeking help for family members [10]. In TikTok, there were more than 200 million users who acquired health information every day. From December 2022 to January 2023, the reading amount of health information–related short videos was more than 45.4 billion [11]. Hence, these short-video sharing platforms played important roles in improving the population's basic knowledge of diseases. However, there is still no strict limitation on the video content and the uploaders on these platforms. Some inaccurate information may be conveyed to the users, leading to misleading and even influencing personal health decisions. Therefore, evaluating the content, accuracy, and quality of the information is essential.

However, to our knowledge, no study has examined information about colorectal polyps on short-video sharing platforms. Therefore, this study aimed to evaluate the quality and reliability of colorectal polyps–related videos on several widely used short-video sharing platforms (TikTok, WeChat, and Xiaohongshu), and then provide some fact-based recommendations for improving the quality and popularity of health-related videos.

## Methods

### Ethical Considerations

No ethics approval was applied for because the study only explored publicly available data on short-video sharing platforms and did not conduct any experiments on human subjects. In addition, there is no identifiable information about individual users or IDs in this study.

### Search Strategy and Data Collection

The search was performed on TikTok, WeChat, and Xiaohongshu between May 26 and June 8, 2024. To minimize the bias introduced by personal recommendation algorithms, we registered a new account, then used “肠息肉” (intestinal polyps) or “结肠息肉” (colonic polyps) or “直肠息肉” (rectal polyps) or “结直肠息肉” (colorectal polyps) or “大肠息肉” (polyps of large intestine) as the keywords to search in above 3 short-video sharing platforms. Previous studies showed that most viewers tend to focus only on the top few pages of search results found online [12,13]. Our study mainly aimed to analyze the colorectal polyps–related health information that most viewers could access from short-video sharing platforms. Therefore, we only collected and recorded the top 100 videos for each search term in different platforms. The inclusion criteria were (1) videos with colorectal polyp content and (2) videos in Chinese. The exclusion criteria were (1) duplicated content, (2) advertisements, and (3) videos with irrelevant contents (polyps in other locations, hemorrhoids, rectal prolapse, etc). For included videos, the characteristics recorded and analyzed were titles, the number of likes, the number of comments, the number of collections, the number of shares, days since upload, video duration, video sources, the number of uploaders’ fans, video presentation forms, and video content.

### Assessment of Content and Quality of Videos

The *Journal of American Medical Association* (JAMA) benchmark criteria is a well-known tool for evaluating the reliability of information obtained from health-related websites, which consists of 4 evaluation dimensions, that is, author, attribution, disclosure, and currency [14,15]. The Global Quality Scale (GQS) is a 5-point Likert scale that can subjectively rate the overall quality of videos and also considers the flow and ease of website usage. It consisted of 5 criteria ranging from 1 point (poor quality) to 5 points (excellent flow and quality) [16,17]. The modified DISCERN is a valid tool and is recommended in the literature as a measure of the reliability and quality in web-based sources [18,19]. It consisted of 5 questions, and each question was given a weight of 1 point. The JAMA score, GQS score, and modified DISCERN score are used extensively in research [20-22]. To further assess the understandability and actionability of these videos, the Patient Education Materials Assessment Tool (PEMAT), developed by the Agency for Healthcare Research and Quality, was also used in this study [23]. The specific scoring criteria of the above score systems are shown in Table S1-S4 in Multimedia Appendix 1.

Scoring was performed by 2 researchers (J-LG and S-HX) independently, and discussions were carried out with a third author (P-YL) to solve any uncertainties. Each video was scored 3 times—twice by J-LG, once by S-HX. This design permitted us to assess interrater and intrarater reliability with the intraclass correlation coefficient (ICC). Reliability was defined as poor (ICC<0.50), moderate (ICC=0.50-0.75), good (ICC=0.75-0.90), or excellent (ICC>0.90) [24]. The 2 researchers had good agreement for the JAMA score (ICC=0.92, 95% CI 0.91-0.93), GQS score (ICC=0.81, 95% CI 0.78-0.83), modified DISCERN score (ICC=0.75, 95% CI 0.72-0.78), understandability (ICC=0.81, 95% CI 0.78-0.83), and actionability (ICC=0.85, 95% CI 0.83-0.87). The intrarater reliability was also good overall (JAMA score=0.98, GQS score=0.85, modified DISCERN score=0.78, understandability=0.78, and actionability=0.86).

### Statistical Analysis

SPSS (version 22.0; IBM Corp) and GraphPad Prism (version 9.0; Dotmatics) were used for analyzing the data. Categorical variables were described as frequencies and percentages. Normally distributed data were presented as a mean (SD), and nonnormally distributed data were expressed as median and range. Comparisons between 2 groups were performed using the Mann-Whitney *U* test or using the Student *t* test, and comparisons among 3 or more groups were performed by the Kruskal-Wallis test or one-way ANOVA. The Spearman correlation analysis was used to evaluate the relationship between quantitative variables. *P* value less than .05 (*P*<.05) was considered statistically significant.

## Results

### The General Characteristics of Videos

**Figure 1:**
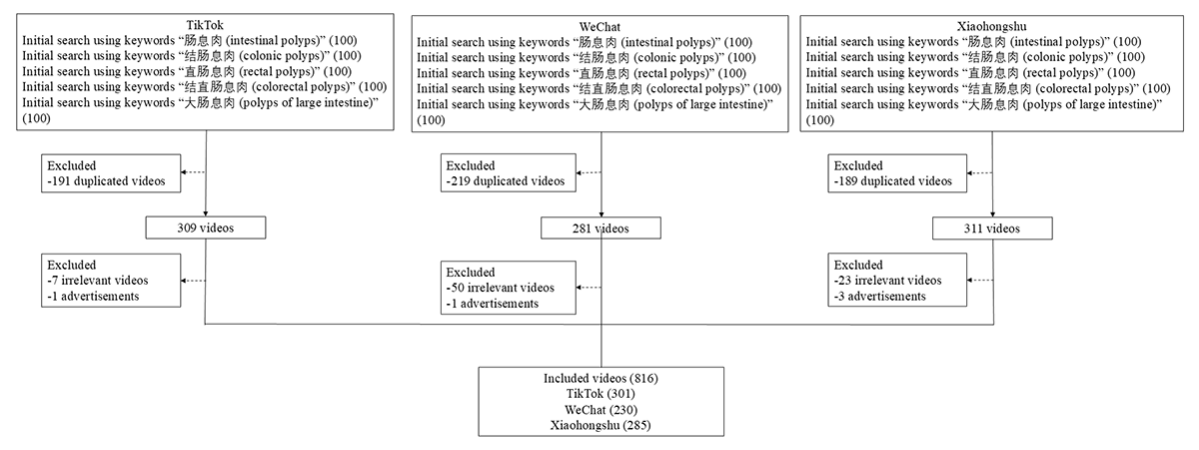
Flowchart of the study.

As shown in Figure 1, after excluding advertisements, duplicated, and irrelevant videos, a total of 816 colorectal polyps–related videos were included for further analysis. Among 816 videos, 301 videos came from TikTok, 230 videos were retrieved from WeChat, and 285 videos came from Xiaohongshu. The number of likes, comments, shares, and collections of videos from TikTok was higher than videos from WeChat and Xiaohongshu (all *P*<.001). The JAMA score, GQS score, and modified DISCERN score of videos from Tiktok and WeChat were higher than videos from Xiaohongshu (mean 2.3, SD 0.5 vs mean 2.2, SD 0.6 vs mean 1.6. SD 0.5; *P*<.001; mean 2.6, SD 0.8 vs mean 2.6, SD 0.8 vs mean 2.3, SD 0.8; *P*<.001; mean 2.9, SD 0.6 vs mean 2.9, SD 0.6 vs mean 1.8, SD 0.7; *P*<.001). There were statistical differences among the understandability and actionability of videos from different platforms (*P*<.001; Table 1). The detailed characteristics of colorectal polyps–related videos in different platforms are shown in Table 1.

**Table 1:** The general characteristics and scores of the colorectal polyps–related videos.

Parameters	Total (N=816)	TikTok (n=301)	WeChat (n=230)	Xiaohongshu (n=285)	*P* value
Duration (seconds), median (range)	59 (5-1417)	72 (5-1417)	67 (6-727)	46 (5-1005)	<.001
Likes, median (range)	55 (0-201,000)	644 (16-201,000)	19 (0-1050)	13 (0-3638)	<.001
Comments, median (range)	5 (0-7205)	52 (0-7205)	1 (0-206)	1 (0-211)	<.001
Collections, median (range)	31 (0-25,000)	149 (1-25,000)	21 (0-3046)	9 (0-3986)	<.001
Shares, median (range)	78 (0-23,000)	128 (0-23,000)	42.5 (0-3168)	—^a^	<.001
Days since upload, median (range)	176 (0-1522)	280 (0-1487)	137 (0-1358)	137 (7-1522)	<.001
Fans of video uploaders, median (range)	13500 (1-62.2×10^5^)	13.2×10^4^ (239-62.2×10^5^)	—	5612 (1-17.7×10^5^)	<.001
JAMA^b^ score, mean (SD)	2.0 (0.6)	2.3 (0.5)	2.2 (0.6)	1.6 (0.5)	<.001
GQS^c^ score, mean (SD)	2.5 (0.8)	2.6 (0.8)	2.6 (0.8)	2.3 (0.8)	<.001
Modified DISCERN score, mean (SD)	2.5 (0.8)	2.9 (0.6)	2.9 (0.6)	1.8 (0.7)	<.001
Understandability, mean (SD)	80.4% (15.6%)	77.8% (17.6%)	84.9% (13%)	79.5% (14.3%)	<.001
Actionability, mean (SD)	42.2% (36.1%)	53.7% (34.4%)	42.6% (37.9%)	30.3% (32.2%)	<.001

^a^Not available.

^b^JAMA: Journal of American Medical Association.

^c^GQS: Global Quality Scale.

### Video Source and Content

Table 2 shows the source and content of videos regarding colorectal polyps. Physicians were the main video uploaders (709/816, 86.9%), and video content mainly included disease knowledge (635/816, 77.8%) and outpatient scenarios (178/816, 21.8%). Disease knowledge in videos mainly focused on treatments (252/816, 30.9%), preventions (157/816, 19.2%), and carcinogenesis (151/816, 18.5%). Expert monologue (546/816, 66.9%) and dialogue (182/816, 22.3%) were dominated in the video presentation forms.

**Table 2:** The sources and content of the colorectal polyps–related videos.

Variable		Total (N=816), n (%)	TikTok (n=301), n (%)	WeChat (n=230), n (%)	Xiaohongshu (n=285), n (%)	*P* value	
**Video source**							<.001
	Physicians	709 (86.9)	294 (97.7)	148 (64.3)	267 (93.7)		
	Hospital	38 (4.7)	1 (0.3)	36 (15.7)	1 (0.4)		
	News agencies	34 (4.2)	3 (1)	31 (13.5)	0 (0)		
	Independent users	26 (3.2)	3 (1)	10 (4.3)	13 (4.6)		
	Others	9 (1.1)	0 (0)	5 (2.1)	4 (1.5)		
**Different medical specialties**							<.001
	TCM^a^ practitioner	352 (49.6)	105 (35.7)	81 (54.7)	166 (62.2)		
	Western medicine practitioner	357 (50.4)	189 (64.3)	67 (45.3)	101 (37.8)		
**Video content**							.006
	Disease knowledge	635 (77.8)	210 (69.8)	200 (87)	225 (78.9)		
	Outpatient scenarios	178 (21.8)	89 (29.6)	30 (13)	59 (20.7)		
	Personal experience	3 (0.4)	2 (0.6)	0 (0)	1 (0.4)		
**Different disease knowledge**							.61
	Treatment	252 (30.9)	81 (26.9)	84 (36.5)	87 (30.5)		
	Prevention	157 (19.2)	49 (16.3)	62 (27)	46 (16.1)		
	Carcinogenesis	151(18.5)	59 (19.6)	47 (20.4)	45 (15.8)		
	Symptom	98 (12)	39 (13)	34 (14.8)	25 (8.8)		
	Definition	45 (5.5)	18 (6)	13 (5.7)	14 (4.9)		
	Posttreatment caveats	41 (5)	18 (6)	12 (5.2)	11 (3.9)		
	Reexamination	40 (4.9)	14 (4.7)	10 (4.3)	16 (5.6)		
	Others	69 (8.5)	20 (6.6)	27 (11.7)	22 (7.7)		
**Video presentation form**							<.001
	Expert monologue	546 (66.9)	183 (60.8)	174 (75.7)	189 (66.3)		
	Dialogue	182 (22.3)	79 (26.2)	36 (15.7)	67 (23.5)		
	Visual pictures and literature	32 (3.9)	13 (4.3)	4 (1.7)	15 (5.3)		
	Endoscope operation demonstration	31 (3.8)	21 (7)	5 (2.2)	5 (1.8)		
	Animation	14 (1.7)	2 (0.7)	7 (3)	5 (1.8)		
	Vlogs of patients	6 (0.7)	2 (0.7)	0 (0)	4 (1.4)		
	Others	5 (0.6)	1 (0.3)	4 (1.7)	0 (0)		

^a^TCM: traditional Chinese medicine.

### The Quality and Popularity of Videos From Different Sources With Different Contents and Different Presentation Forms

**Figure 2:**
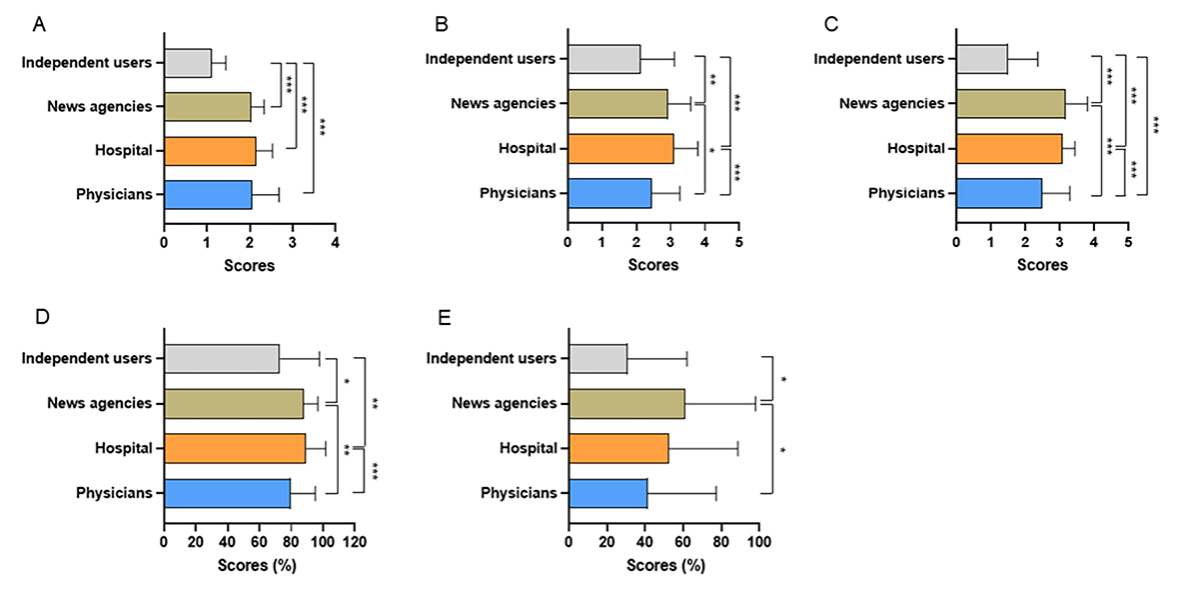
The Journal of American Medical Association (JAMA) score, Global Quality Scale (GQS) score, modified DISCERN score, Patient Education Materials Assessment Tool (PEMAT)–understandability, and PEMAT-actionability of videos on colorectal polyps from different sources. (A) the JAMA score, (B) the GQS score, (C) the modified DISCERN score, (D) PEMAT-understandability, and (E) PEMAT-actionability. *P<.05, **P<.01, ***P<.001.

**Figure 3:**
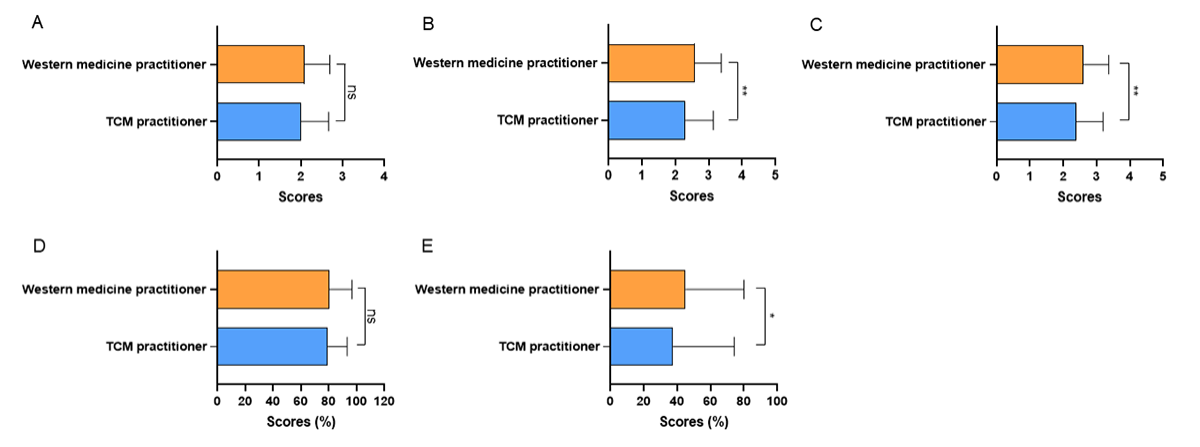
The Journal of American Medical Association (JAMA) score, Global Quality Scale (GQS) score, modified DISCERN score, Patient Education Materials Assessment Tool (PEMAT)–understandability, and PEMAT-actionability of videos on colorectal polyps of Western medicine practitioners and traditional Chinese medicine practitioners. (A) the JAMA score, (B) the GQS score, (C) the modified DISCERN score, (D) PEMAT-understandability, (E) PEMAT-actionability. ns: not significant; TCM: traditional Chinese medicine. *P<.01, **P<.001.

**Figure 4:**
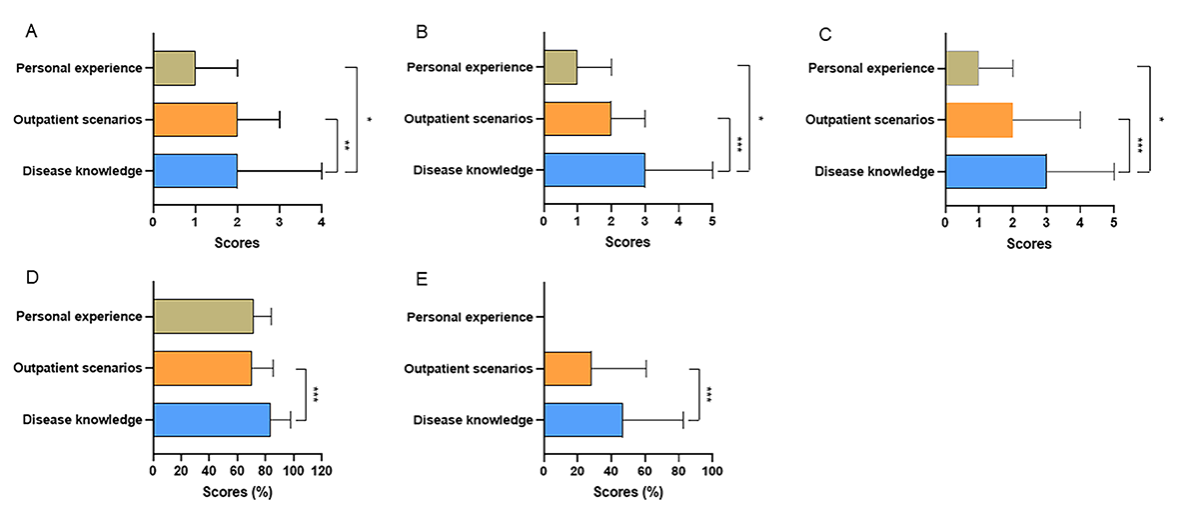
The Journal of American Medical Association (JAMA) score, Global Quality Scale (GQS) score, modified DISCERN score, Patient Education Materials Assessment Tool (PEMAT)–understandability, and PEMAT-actionability of videos on colorectal polyps with different contents. (A) the JAMA score, (B) the GQS score, (C) the modified DISCERN score, (D) PEMAT-understandability, and (E) PEMAT-actionability. *P<.05, **P<.01, ***P<.001.

The JAMA scores and modified DISCERN scores of videos uploaded by independent users were significantly lower than videos uploaded by physicians’ hospitals and news agencies (all *P<*.001; Figures 2A and 2C). The GQS score and understandability of videos uploaded by hospitals and news agencies were higher than those uploaded by physicians (*P*<.001, *P*<.001, *P*=.01, and *P*=.009, respectively) and independent users (*P*<.001, *P*=.004, *P*<.001, and *P*=.03, respectively; Figures 2B and 2D). In addition, the actionability of videos uploaded by news agencies was better than physicians and independent users (*P*=.02 and *P*=.01; Figure 2E). Among videos uploaded by physicians, the GQS score, modified DISCERN score, and actionability of videos uploaded by traditional Chinese medicine (TCM) practitioners were lower than videos uploaded by Western medicine practitioners (*P<*.001 and *P*<.001, and *P*=.006, respectively), while there were no significant differences in the JAMA score and understandability between the 2 groups (Figures 3A-3E). The JAMA score, GQS score, modified DISCERN score, understandability, and actionability of videos about disease knowledge were significantly higher than videos about outpatient scenarios (*P<*.001, *P*=.02, *P*<.001, *P*<.001, and *P*<.001, respectively; Figures 4A-4E).

**Figure 5:**
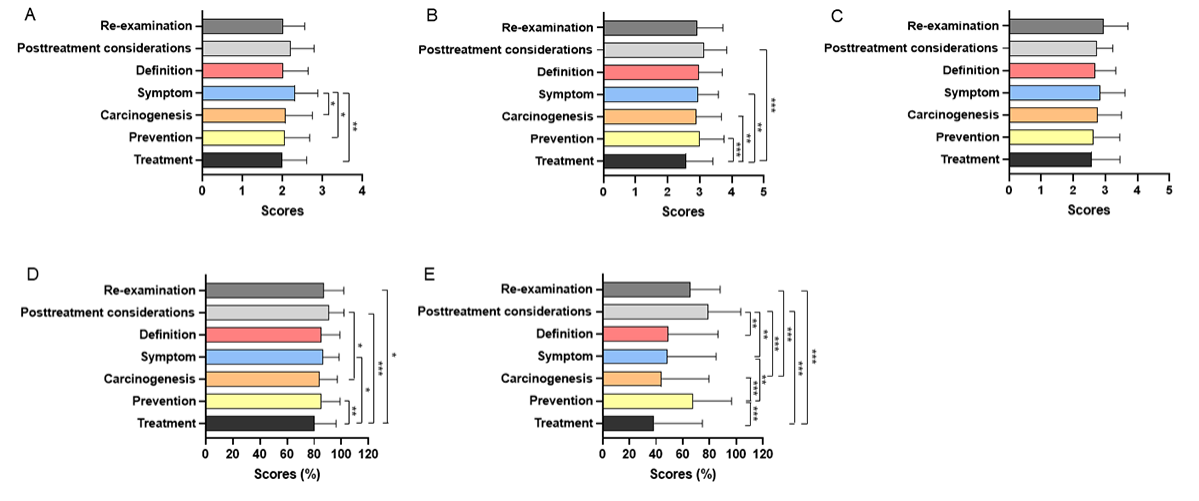
The Journal of American Medical Association (JAMA) score, Global Quality Scale (GQS) score, modified DISCERN score, Patient Education Materials Assessment Tool (PEMAT)–understandability, and PEMAT-actionability of videos on colorectal polyps with different disease knowledge. (A) the JAMA score, (B) the GQS score, (C) the modified DISCERN score, (D) PEMAT-understandability, and (E) PEMAT-actionability. *P<.05, **P<.01, ***P<.001.

**Figure 6:**
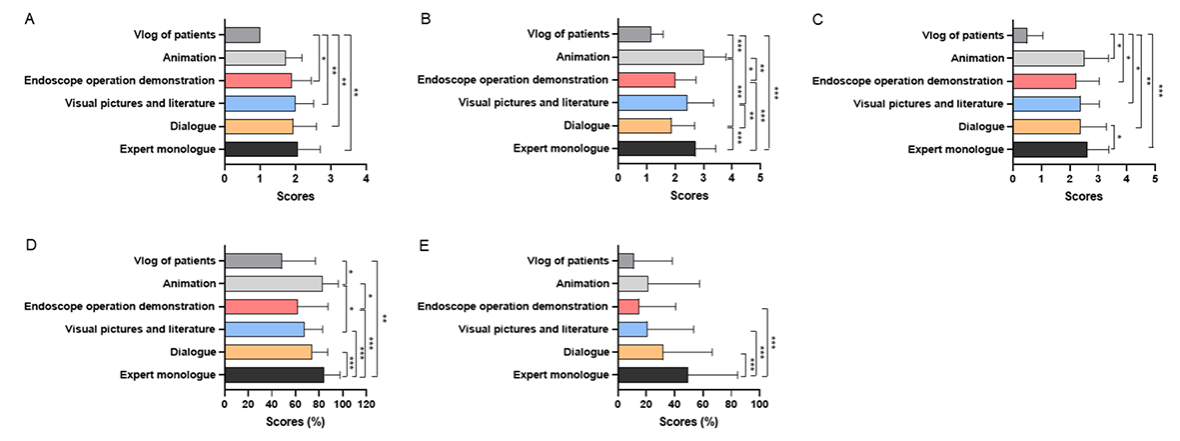
The Journal of American Medical Association (JAMA) score, Global Quality Scale (GQS) score, modified DISCERN score, Patient Education Materials Assessment Tool (PEMAT)–understandability, and PEMAT-actionability of videos on colorectal polyps with different presentation forms. (A) the JAMA score, (B) the GQS score, (C) the modified DISCERN score, (D) PEMAT-understandability, and (E) PEMAT-actionability. *P<.05, **P<.01, ***P<.001.

Among videos about disease knowledge, videos about symptoms had higher JAMA scores than videos about treatment, prevention, and carcinogenesis (*P*=.001, *P*=.02, and *P*=.048, respectively; Figure 5A). Compared with other disease knowledge, videos about treatment had lower GQS scores than prevention, carcinogenesis, symptom, and posttreatment caveats (*P*<.001, *P*=.003, *P=*.001, and *P*<.001, respectively; Figure 5B). There was no significant difference in the modified DISCERN score among videos about different disease knowledge (Figure 5C). Meanwhile, videos conveying treatment had poorer understandability than those conveying prevention, symptoms, posttreatment caveats, and reexamination (*P*=.005, *P*=.02, *P*=.04, and *P*<.001, respectively). As for actionability, videos about prevention, posttreatment caveats, and reexamination were better than those about treatment (*P*<.001, *P*<.001, and *P*=.001) and carcinogenesis (*P*<.001, *P*<.001, and *P*=.04; Figures 5D and 5E). Among different video presentation forms, videos presented in vlogs of patients had lower JAMA scores and modified DISCERN scores. Videos presented in expert monologues or animation had higher GQS scores than those presented in dialogue (*P*<.001 and *P*<.001), endoscope operation demonstration (*P*<.001 and *P*=.006), and vlogs (*P*<.001 and *P*=.001). In addition, the understandability of the forms of expert monologues and animation were better than the forms of endoscope operation demonstration (*P*<.001 and *P*=.02), visual pictures and literature (*P*<.001 and *P*=.01), and vlogs (*P*=.004 and *P*=.03). The form of expert monologues had better actionability than dialogue (*P*<.001), endoscope operation demonstration (*P*<.001), visual pictures and literature (*P*<.001; Figures 6A-6E).

We also compared the popularity (likes, comments, collections, and shares) of videos uploaded by different sources with different contents and different presentation forms (Table 3). Among different video sources, physicians, especially Western medicine practitioners, received more likes and comments (*P*≤.001). Videos conveying posttreatment caveats and reexamination were more likely to be collected and shared (*P*=.03 and *P*=.006).

**Table 3:** The popularity of videos from different sources with different contents and different presentation forms.

Variables		Likes	Comments	Collections	Shares
**Video source**					
	Physician, mean (range)	64 (0-201,000)	6 (0-7205)	33 (0-25,000)	83 (0-23,000)
	Hospital, mean (range)	23 (0-4019)	0 (0-177)	24 (0-1227)	38 (1-934)
	News agency, mean (range)	62 (0-3744)	3 (0-109)	32 (1-1246)	114 (1-2842)
	Independent user, mean (range)	13 (0-536)	3 (0-383)	15 (0-288)	29 (4-110)
	Others, mean (range)	11 (1-71)	0 (0-12)	9 (2-320)	52 (15-649)
	*P* value	.001	.001	.06	.81
**Different medical specialties**					
	TCM^a^ practitioner, mean (range)	27 (0-35,000)	3 (0-1692)	21 (0-7863)	73 (1-5828)
	Western medicine practitioner, mean (range)	152 (0-201,000)	13 (0-7205)	54 (0-25,000)	90 (0-23,000)
	*P* value	<.001	<.001	<.001	.06
**Video content**					
	Disease knowledge, mean (range)	52 (0-201,000)	4 (0-7205)	33 (0-25,000)	89 (0-23,000)
	Outpatient scenarios, mean (range)	70 (0-27,000)	11 (0-1692)	27 (0-2582)	50 (1-5100)
	Personal experience, mean (range)	95 (3-186)	37 (0-73)	8 (1-15)	5 (5-5)
	*P* value	.38	.02	.16	.02
**Different disease knowledge**					
	Treatment, mean (range)	50 (0-201,000)	4 (0-7205)	31 (0-25,000)	74 (1-23,000)
	Prevention, mean (range)	68 (0-139,000)	4 (0-4752)	39 (0-20,000)	125 (0-23,000)
	Carcinogenesis, mean (range)	60 (0-201,000)	4 (0-7205)	35 (0-25,000)	90 (0-23,000)
	Symptom, mean (range)	58 (0-43,000)	4 (0-1197)	29 (0-7637)	61 (2-4947)
	Definition, mean (range)	63 (0-5857)	3 (0-1146)	28 (0-1962)	54 (0-4947)
	Posttreatment caveats, mean (range)	106 (2-32,000)	10 (0-1834)	94 (1-11,000)	200 (10-20,000)
	Reexamination, mean (range)	80 (1-139,000)	6 (0-4752)	70 (0-20,000)	117 (0-23,000)
	Others, mean (range)	47 (0-7897)	3 (0-1146)	26 (0-1686)	51 (1-4947)
	*P* value	.25	.31	.03	.006
**Video presentation form**					
	Expert monologue, mean (range)	50 (0-108,000)	4 (0-4362)	33 (0-14,000)	82 (0-20,000)
	Dialogue, mean (range)	55 (0-201,000)	8 (0-7205)	27 (0-25,000)	56 (1-23,000)
	Endoscope operation demonstration, mean (range)	340 (2-3314)	42 (0-1416)	93 (1-584)	120 (2-1140)
	Visual pictures and literature, mean (range)	78 (1-13,000)	4 (0-1305)	43 (0-3986)	91 (3-2409)
	Animation, mean (range)	12 (0-949)	0 (0-211)	15 (0-364)	54 (1-249)
	Vlog of patients, mean (range)	14 (0-536)	9 (0-383)	4 (0-48)	35 (5-65)
	Others, mean (range)	66 (13-177)	4 (2-7)	28 (12-73)	49 (18-121)
	*P* value	.02	<.001	.048	.39

^a^TCM: traditional Chinese medicine.

### Correlation Analysis

Spearman correlation (ρ) analysis revealed the relationship among different video variables. It was shown that there were positive correlations among the number of likes, comments, collections, shares, and fans (all *P*<.001), while the duration of videos was not associated with the number of likes, comments, collections, and shares (all *P*>.05; Table 4). The number of likes, comments, collections, and fans was positively correlated with the JAMA score, GQS score, and modified DISCERN score. Videos with higher GQS scores and modified DISCERN scores were associated with more shares (*P*<.001 and *P*<.001, respectively) and longer duration (*P*<.001 and *P*=.04, respectively). Actionability was positively correlated with the number of collections, shares, fans, and longer duration (*P*=.003, *P*=.03, *P*<.001, and *P*<.001, respectively), while understandability had a negative correlation with the duration of videos (*P*=.001; Table 5).

**Table 4:** The correlation analysis between the video variables.

Variables		Likes	Comments	Collections	Shares
**Likes**					
	ρ	1	0.898	0.939	0.810
	*P* value	—^a^	<.001	<.001	<.001
**Comments**					
	ρ	0.898	1	0.861	0.840
	*P* value	<.001	—	<.001	<.001
**Collections**					
	ρ	0.939	0.861	1	0.885
	*P* value	<.001	<.001	—	<.001
**Shares**					
	ρ	0.810	0.840	0.885	1
	*P* value	<.001	<.001	<.001	—
**Duration**					
	ρ	0.032	0.036	0.054	0.020
	*P* value	.40	.31	.13	.64
**Days since uploaded**					
	ρ	0.048	0.113	0.046	0.101
	*P* value	.17	.001	.19	.02
**Fans**					
	ρ	0.442	0.439	0.436	0.410
	*P* value	<.001	<.001	<.001	<.001

^a^Not applicable.

**Table 5:** The correlation analysis between video variables and the video quality.

Variables		JAMA^a^	GQS^b^	Modified DISCERN	Understandability	Actionability
**Likes**						
	ρ	0.118	0.135	0.167	–0.006	0.057
	*P* value	.001	<.001	<.001	.85	.10
**Comments**						
	ρ	0.102	0.107	0.189	–0.036	0.053
	*P* value	.004	.002	<.001	.30	.13
**Collections**						
	ρ	0.125	0.158	0.174	–0.016	0.106
	*P* value	<.001	<.001	<.001	.65	.003
**Shares**						
	ρ	0.055	0.185	0.177	0.012	0.093
	*P* value	.20	<.001	<.001	.78	.03
**Duration**						
	ρ	0.042	0.247	0.073	–0.116	0.168
	*P* value	.23	<.001	.04	.001	<.001
**Fans**						
	ρ	0.182	0.127	0.269	0.001	0.149
	*P* value	<.001	.002	<.001	.98	<.001

^a^JAMA: Journal of American Medical Association.

^b^GQS: Global Quality Scale.

## Discussion

### Principal Findings

Health problems are crucial concerns in our daily lives, which require significant attention, accurate assessment, and prompt intervention. With the increasing popularity of mobile internet, it has become one of the most popular ways for obtaining health and medical information. A survey showed that 70% of network users first acquired health information from the internet [25]. There have been studies that evaluated the quality of health information on several diseases, like gallstone disease, chronic obstructive pulmonary disease, and diabetes on the internet. It was found that the quality of these popular science videos on different diseases varied significantly [17,26,27]. So, how about the reliability and quality of colorectal polyps–related videos online? In this study, we analyzed the content, reliability, and quality of videos regarding colorectal polyps from 3 popular short-video sharing platforms—TikTok, WeChat, and Xiaohongshu. Among these, videos on TikTok were more popular and received more likes, comments, collections, and shares. Notably, the overall quality of short videos related to colorectal polyps was unsatisfactory, especially videos from Xiaohongshu. Physicians were the main uploaders, and most videos aimed to convey disease knowledge directly in the form of expert monologue. Disease knowledge about treatments, preventions, and carcinogenesis were the most frequently discussed in colorectal polyps–related videos. In addition, videos from physicians and news agencies were heavily preferred, and videos from news agencies and hospitals had higher quality. As for video content, videos primarily concerned with disease knowledge are more popular and of higher quality.

### Factors Correlated With the Popularity of Videos

The number of likes of videos on colorectal polyps attained 201,000 at most, and the number of collections and shares of some videos was more than 10,000, which showed that colorectal polyps have drawn much attention. It was reported that the number of likes, comments, collections, and shares could partly reflect the popularity of videos [28,29]. In this study, we found that videos uploaded by Western medicine practitioners and TCM practitioners were almost in equal numbers, but videos from Western medicine practitioners were more popular. It was possibly because the current prominence of the Western medicine may result in people having fewer opportunities to be exposed to TCM, especially for young people, the main users of the internet. The current study also showed that most videos from short-video sharing platforms aimed to convey disease knowledge, and videos about posttreatment caveats and reexamination were more likely to be collected and shared by viewers. It was reported that the risk of bleeding and perforation increased approximately 7 times among those who had a polypectomy compared with those who did not [30]. Hence, knowing the caveats after polypectomy is conducive to postoperative recovery and to avoid adverse outcomes. A meta-analysis confirmed the risk of recurrence of colorectal polyps after standard endoscopic mucosal resection [31], and some of them eventually develop into CRC [4,32]. However, many patients ignore the importance of reexamination in clinical practice. In addition, in the case of different types of polyps, the time interval for reexamination may change. Videos about reexamination could inform patients about the importance of reexamination and instruct them on how to reexamine. Furthermore, there was a positive correlation between likes, comments, collections, and shares, which indicated that a video receiving more likes and comments would be more likely to be collected and shared. The number of fans played an important role in the popularity of videos, thus the influence of these uploaders with large numbers of fans cannot be ignored in conveying health-related information.

### Factors Correlated With Video Quality

Although the majority of videos regarding colorectal polyps were uploaded by physicians and mainly conveyed disease knowledge, the overall reliability and quality of these videos regarding colorectal polyps were not good enough. This was consistent with several previous studies. For example, Lee et al [33] analyzed the videos regarding gallstone disease on YouTube (Google) and were surprised to find that more than half of the videos were misleading and presented a risk of harmful consequences. Kong et al [27] assessed the quality of the information in diabetes-related videos on TikTok and found that the overall quality of the information from these videos did not fully meet the health information needs of patients with diabetes. In this study, we also found that a part of uploaders claimed to be medical staff, but did not authenticate their identities. It may be possible that some uploaders pretend to be physicians to increase trust and video views, which affected the overall reliability of colorectal polyps–related videos. In addition, some uploaders may convey inaccurate information or exaggerate the effect of treatment for building a more professional image. However, incorrect health information in short videos may increase patients’ risks who may make health decisions based on inaccurate information. Hence, people should exercise caution when acquiring health information online.

The quality of videos uploaded by news agencies and hospitals were higher than those uploaded by physicians. These results are compatible with some studies evaluating health information videos [15,34]. This could be because of videos from the official accounts of institutions are in general created by professional media personnel with the participation of medical professionals. It is noteworthy that video duration demonstrated a positive correlation with video quality, which was consistent with previous studies [21,35,36]. Indeed, half of the videos in our study lasted for less than 1 minute, even a few or a dozen seconds, making it difficult to provide sufficient information, which may greatly affect the video quality. Also, video presentation forms were associated with the quality of videos. Compared with dialogue and vlogs, expert monologue could convey more health information in a short time, and animation could make disease knowledge easy to understand. In addition, the number of likes, shares, and comments was positively correlated with the quality of videos. That is to say, the reliability and substantiality of video contents are important factors that increase the popularity of the videos.

### Recommendations Based on Our Results

There is no doubt that health-related content is more complicated, professional, and less attractive than other entertainment videos, therefore it is a challenge to attract more viewers. To increase the influence of related videos, choosing physicians and news agencies with large numbers of fans to further spread correct medical information may be a good idea. Furthermore, building up a medical classification module could further assist users in accessing health information easily. Also, providing standardized criteria for medical videos on short-video sharing platforms, and hiring professional medical workers to conduct reviews before uploading the videos could improve the quality and reliability of health information. In addition, platform administrators need to enhance the management of identity authentication to increase the users’ recognition and dissemination of professional information. Videos that are very short cannot provide abundant information, while very long videos may increase the difficulty of understanding. Hence, video uploaders need to think about how to offer adequate information of high quality within a reasonable duration. On the other hand, videos on how to treat colorectal polyps occupied a large proportion, but the quality was quite unsatisfactory and urged to be improved.

### The Significance of This Study

Improving the public’s general understanding of colorectal polyps can aid in earlier recognition, prevention, and management of colorectal polyps and CRC. Short videos on the internet are important sources for the public to access health-related information, particularly in a medical resource–limited setting. However, the quality varies across short videos containing knowledge about different diseases. This is the first study to evaluate the quality and reliability of short videos regarding colorectal polyps using multiple tools (JAMA score, GQS score, modified DISCERN score, and PEMAT). Our study analyzed the popularity, and the quality of different sources, contents, and presentation forms of videos regarding colorectal polyps. We found that the quality of related videos needed to be improved. Based on the results, we provided some suggestions on how to improve the popularity and quality of short videos regarding colorectal polyps. It would be helpful to produce the right, reliable, and high-quality information source and improve the public’s understanding of colorectal polyps.

### Limitations

There are some limitations in this study. First, the video search was only performed on Chinese video-sharing platforms, so the findings may not apply to platforms in other languages. Second, only the top 100 videos of each search term rather than all searched videos were included, which may not reflect the overall situation. But most users usually only view the first few pages of search results, our results may be more in line with the information acquired by most users. Third, the present scoring criteria of video quality are mainly based on subjective judgment. Although scoring was performed independently by 2 researchers and they had good agreement, subjective differences still could not be ignored. Finally, although we registered and logged in new accounts on each platform, the bias in the selection and evaluation of the videos may still have existed.

### Conclusion

Colorectal polyps–related videos on short-video sharing platforms were mainly uploaded by physicians and mainly conveyed information regarding disease knowledge in the form of an expert monologue. However, the overall reliability and quality of these videos were not good enough and need to be improved. Users should remain cautious and selective when watching videos regarding colorectal polyps on short-video sharing platforms.

## Appendix

Multimedia Appendix 1 Additional tables.

## Abbreviations

CRC: colorectal cancer
GQS: Global Quality Scale
ICC: intraclass correlation coefficient
JAMA: Journal of American Medical Association
PEMAT: Patient Education Materials Assessment Tool
TCM: traditional Chinese medicine
