# Supplementary material for: Videos in Short-Video Sharing Platforms as Sources of Information on Colorectal Polyps: Cross-Sectional Content Analysis Study
Source: J Med Internet Res. 2024 Oct 29;26:e51655. doi: 10.2196/51655 (PMC11558218; doi:10.2196/51655)
Supplement: Multimedia Appendix 1 [file jmir_v26i1e51655_app1.docx]

**Table S1. The *Journal of American Medical Association* (JAMA) benchmark criteria.**

| Criteria (1 point for each) | Description |
| --- | --- |
| Authorship | Author and contributor credentials and their affiliations should be provided |
| Attribution | Clearly lists all copyright information and states references and sources for content |
| Currency | Initial date of posted content and subsequent updates to content should be provided |
| Disclosure | Conflicts of interest, funding, sponsorship, advertising, support, and video ownership should be fully disclosed |

**Table S2. Description of the Global Quality Score (GQS) scale.**

| Scale | Description |
| --- | --- |
| Poor quality (1 point) | Poor quality and poor flow of the site, most information missing, not at all useful for patients |
| Generally poor quality (2 point) | Generally poor quality and poor flow, some information listed but many important topics missing, of very limited use to patients |
| Moderate quality (3 point) | Moderate quality, sub-optimal flow, some important information is adequately discussed but others poorly discussed, somewhat useful for patients |
| Good quality (4 point) | Good quality and generally good flow, most of the relevant information is listed, but some topics not covered, useful for patients |
| Excellent quality (5 point) | Excellent quality and excellent flow, very useful for patients |

**Table S3. Description of the modified DISCERN score.**

| Criteria  (1 point for each) | Description |
| --- | --- |
| 1 | Is the video clear, concise, and understandable? |
| 2 | Are reliable sources of information used? (i.e., publication cited, speaker is specialist) |
| 3 | Is the information presented balanced and unbiased? |
| 4 | Are additional sources of information listed for patient reference? |
| 5 | Are areas of uncertainty/controversy mentioned? |

**Table S4. The Patient Education Materials Assessment Tool (PEMAT)**

**Understandability.**

|  | Item | Response Options | Rating |
| --- | --- | --- | --- |
| Topic: Content | | | |
| 1 | The material makes its purpose completely evident. | Disagree=0, Agree=1 |  |
| Topic: Word Choice & Style | | | |
| 3 | The material uses common, everyday language. | Disagree=0, Agree=1 |  |
| 4 | Medical terms are used only to familiarize audience with the terms. When used, medical terms are defined. | Disagree=0, Agree=1 |  |
| 5 | The material uses the active voice. | Disagree=0, Agree=1 |  |
| Topic: Organization | | | |
| 8 | The material breaks or "chunks" information into short sections. | Disagree=0, Agree=1,  Very short material=N/A |  |
| 9 | The material’s sections have informative headers. | Disagree=0, Agree=1,  Very short material=N/A |  |
| 10 | The material presents information in a logical sequence. | Disagree=0, Agree=1 |  |
| 11 | The material provides a summary. | Disagree=0, Agree=1,  Very short material=N/A |  |
| Topic: Layout & Design | | | |
| 12 | The material uses visual cues (e.g., arrows, boxes, bullets, bold, larger font, highlighting) to draw attention to key points. | Disagree=0, Agree=1, Video=N/A |  |
| 13 | Text on the screen is easy to read. | Disagree=0, Agree=1,  No text or all text is narrated=N/A |  |
| 14 | The material allows the user to hear the words clearly (e.g., not too fast, not garbled). | Disagree=0, Agree=1,  No narration=N/A |  |
| Topic: Use of Visual Aids | | | |
| 18 | The material uses illustrations and photographs that are clear and uncluttered. | Disagree=0, Agree=1,  No visual aids=N/A |  |
| 19 | The material uses simple tables with short and clear row and column headings. | Disagree=0, Agree=1,  No tables=N/A |  |

Total Points: _____________

Total Possible Points: _____________

Understandability Score (%): _____________

(Total Points / Total Possible Points x 100)

**Actionability**

|  | Item | Response Options | Rating |
| --- | --- | --- | --- |
| 20 | The material clearly identifies at least one action the user can take. | Disagree=0, Agree=1 |  |
| 21 | The material addresses the user directly when describing actions. | Disagree=0, Agree=1 |  |
| 22 | The material breaks down any action into manageable, explicit steps. | Disagree=0, Agree=1 |  |
| 25 | The material explains how to use the charts, graphs, tables, or diagrams to take actions. | Disagree=0, Agree=1,  No charts, graphs, tables, diagrams=N/A |  |

Total Points: _____________

Total Possible Points: _____________

Actionability Score (%): _____________

(Total Points / Total Possible Points x 100)
